# Development and optimization of an easy to interpret loop-mediated isothermal amplification (LAMP) assay for the identification of bacterial pathogens causing childhood pneumonia

**DOI:** 10.3389/fmicb.2026.1748456

**Published:** 2026-03-02

**Authors:** Arturo Martínez-Trejo, Andrea Vergara, Giulia Gatti, Elisabet Guiral, Jorge Otero, Alba Sánchez, Anna Rull, Olga Calavia, Andrea Papaleo, Ramón Farré, Jordi Vila

**Affiliations:** 1 Barcelona Institute for Global Health (ISGlobal), Barcelona, Spain; 2 School of Medicine and Health Sciences, University of Barcelona, Barcelona, Spain; 3 Department of Clinical Microbiology, CDB, Hospital Clínic of Barcelona, Barcelona, Spain; 4 Centro de Investigación Biomédica en Red (CIBER) de Enfermedades Infecciosas (CIBERINFEC), Instituto Salud Carlos III, Madrid, Spain; 5 Department of Surgical & Medical Sciences-DIMEC, Alma Mater Studiorum-University of Bologna, Bologna, Italy; 6 Unit of Biophysics and Bioengineering, School of Medicine and Health Sciences, University of Barcelona, Barcelona, Spain; 7 Centro de Investigación Biomédica en Red (CIBER) de Enfermedades Respiratorias (CIBERES), Madrid, Spain; 8 Institut de Recerca Biomèdica Catalunya Sud (formerly IISPV), Hospital Universitari Joan XXIII de Tarragona, Universitat Rovira I Virgili (URV), Tarragona, Spain

**Keywords:** childhood pneumonia, diagnostic tool, HNB/SYTO 9 dual-system, LAMP, loop-mediated isothermal amplification, molecular diagnosis, pneumonia-causing bacteria

## Abstract

**Introduction:**

Pneumonia remains the leading infectious cause of death in children under five, especially in low-resource settings. Reducing mortality requires rapid, accessible, and reliable diagnostic tools. In this regard, the loop-mediated isothermal amplification (LAMP) technique has emerged as a fast and efficient alternative for simple pathogen detection. This study aimed to standardise and optimize a LAMP assay for detecting the main bacteria causing pneumonia in children, including *Streptococcus pneumoniae*, *Staphylococcus aureus*, *Haemophilus influenzae*, *Klebsiella pneumoniae*, and *Mycoplasmoides pneumoniae* using a simple visual readout.

**Methods:**

Several fluorescent and colorimetric dyes were evaluated to identify those providing a clear readout visible to the naked eye. Once achieved, detection conditions for each pathogen in the panel were optimized, and the feasibility of the assay was assessed using respiratory clinical samples, including both confirmed positives and negatives for the bacteria targeted in the panel.

**Results and discussion:**

SYBR Safe, Calcein-Mn^2+^, and SYTO 9 alone did not show a clear differentiation between positive and negative reactions. In contrast, the combination of hydroxynaphthol blue (HNB) and SYTO 9 proved suitable, providing a clear visual readout to the naked eye after optimization of concentrations and reaction conditions. The selected concentrations were 341.25 μM HNB and 0.75 μM SYTO 9, which enabled clear and stable fluorescence-based visualization of LAMP results, remaining visible for several months. The technique showed low detection limits: 3.9 ×10^3^ CFU/mL for *S. pneumoniae*, 1.7 ×10^5^ CFU/mL for *S. aureus*, 8.2 ×10^3^ CFU/mL for *H. influenzae,* and 1.27 ×10^3^ genome copies/reaction for *M. pneumoniae*. Primers designed to detect *K. pneumoniae* had high specificity and no cross-reactivity with a sensitivity of 1.5 × 10^4^ CFU/mL. Detection times over 45**–**50 min may suggest colonization instead of active infection. The evaluation of the technique using clinical samples demonstrated its potential feasibility and applicability in real-world clinical settings. Although standardized under laboratory conditions, this LAMP technique shows promise for detecting major pneumonia-causing bacteria in children and could be particularly valuable in low-resource settings. Its rapid, sensitive, and affordable nature may help improve diagnostics and reduce pneumonia-related mortality. However, larger clinical validation studies are needed to confirm its performance and real-world applicability.

## 1 Introduction

Pneumonia is a major global disease and the leading infectious cause of death in children under five disease according to the World Health Organisation (WHO) (Pneumonia in children, 2022). In 2019, the WHO and the United Nations International Children’s Emergency Fund (UNICEF) estimated that 84 children die from pneumonia every hour, mostly in sub-Saharan Africa and South Asia (Pneumonia in children, 2022; Pneumonia in children: What you need to know | UNICEF, 2024). The actual burden is likely underestimated due to unreported cases.

Pneumonia is caused by bacteria, viruses, and fungi. According to the WHO, the main pathogens are *Streptococcus pneumoniae*, *Haemophilus influenzae*, *respiratory syncytial virus* (RSV), and *Pneumocystis jirovecii* (Pneumonia in children, 2022). While pathogen prevalence varies depending on factors such as geography and vaccination coverage, several remain consistently common worldwide, including *S. pneumoniae*, RSV, rhinovirus (HRV), human metapneumovirus (hMPV), influenza viruses, *Moraxella catarrhalis*, and *Mycoplasmoides pneumoniae* (Bénet et al., 2017; Pneumonia Etiology Research for Child Health (PERCH) Study Group, 2019). Pneumonia in children: What you need to know | UNICEF (2024) study of pneumonia-related death in South Asia and sub-Saharan Africa found *S. pneumoniae*, *Klebsiella pneumoniae*, and *H. influenzae* as the primary pathogens (Mahtab et al., 2024).

The timely and accurate identification of the causative pathogen in childhood pneumonia is essential to guide appropriate treatment and reduce associated mortality. Each hour of treatment delay increases the risk of death (Jones et al., 2020). While bacterial culture is the gold standard, it takes 24**–**72 h. PCR-based methods are faster but costly and require complex equipment, limiting their use in high-burden regions. Thus, alternative diagnostic methods must be rapid, reliable, and accessible where necessary.

Molecular techniques like loop-mediated isothermal amplification (LAMP) offer promising alternatives to conventional methods and have been widely used in recent years for pathogen detection (Mori and Notomi, 2009; Atceken et al., 2023). First described in 2000 (Notomi, 2000). LAMP can detect specific DNA and RNA sequences at a constant temperature, making it simpler and more accessible than PCR, as it requires no complex equipment. Results can be obtained in less than an hour, with performance comparable to, or even better than, PCR (Foo et al., 2020; Inaba et al., 2021).

A key advantage of this technology is the simplicity of distinguishing positive from negative reactions. This can be done by measuring turbidity due to the large amount of products (Yuan et al., 2019), or by a simple electrophoresis to observe a characteristic amplification banding pattern (Carvajal-Gamez et al., 2024).

Fluorescent and colorimetric dyes are widely used in LAMP reactions. During amplification, pH indicators, including phenol red, neutral red, and cresol red, undergo a colour change during amplification (Huang et al., 2020). Other dyes, like malachite green and leuco-crystal violet, change their emitted colour upon interaction with LAMP products (Gachugia et al., 2020; Martín-Ramírez et al., 2022). Metal ion indicators like hydroxynaphthol blue (HNB), calcein, and Eriochrome black T produced visible colour changes perceived by the naked eye, through Mg^2+^ binding (Logeshwari et al., 2022; Park, 2022). Some indicators, including calcein and HNB, exhibit fluorescence when exposed to specific excitation wavelengths as well (Fischbach et al., 2015; Li et al., 2023).

Fluorescent dyes include DNA-intercalating compounds, such as SYBR Green I, Eva Green, Berberine, SYTO family (e.g., SYTO 9, 16, 13, 82, 81, and 84) (Fischbach et al., 2015; Quyen et al., 2019; Ruang-areerate et al., 2022). Some LAMP assays also use fluorophore-labelled primers that release fluorescence during amplification (Crego-Vicente et al., 2024).

LAMP is a promising diagnostic tool for pneumonia, offering high sensitivity and specificity, ease of use, low cost, and ease of result interpretation, making it suitable for point-of-care (POC) diagnostic (Soroka et al., 2021). Although LAMP has shown promise in detecting respiratory (Kang et al., 2012; Vergara et al., 2020; Lee et al., 2022), no existing LAMP-based screening panels currently target the most common bacterial causes of childhood pneumonia using a simple, easy-to-interpret assay. Most existing studies still rely on complex and expensive PCR-based equipment.

This study aimed to standardise an accessible, robust, and visually interpretable LAMP-based detection method for the direct identification of the main bacterial pathogens causing childhood pneumonia (*S. pneumoniae*, *Staphylococcus aureus*, *H. influenzae*, *K. pneumoniae*, and *M. pneumoniae*), without requiring advanced equipment or complex sample pre-treatment.

## 2 Materials and methods

### 2.1 Selection of the optimal dye combination for visual detection of LAMP results

The reaction was prepared using Isothermal Amplification Buffer Pack 10X, Magnesium Sulphate Solution (MgSO_4_) 100 mM, Bst 2.0 Warm Start Enzyme 8,000 U/mL, Deoxynucleotide (dNTP) Solution Mix 8 μmol, all from New England Biolabs (Ipswich, MA, USA) and Betaine Solution 5 M from Sigma Aldrich (St. Louis, MO, USA), as base reagents for the reaction. The base concentrations are detailed in Supplementary Table S1.

To determine the optimal visual distinction between positive and negative LAMP reactions, individual dyes, including SYBR Safe, Calcein-Mn, and SYTO 9, were tested separately to assess their performance. Subsequently, the combination of HNB and SYTO 9 was explored. This allowed the selection of the optimal dye system for visual detection in the LAMP assay.

One of the simplest methods to differentiate positive from negative LAMP reactions is the use of intercalating double strand-DNA (dsDNA) agents such as SYBR Safe. We tested this compound as dye; for that, we used SYBR Safe DNA Gel Stain 10,000X in DMSO from Invitrogen (Waltham, MA, USA), testing final concentrations of 16X, 3.2X, 1.6X, and 1X in the LAMP reaction using DMSO as diluent (Thita et al., 2019).

The use of calcein combined with Mn^2+^ ions (Calcein-Mn) is a well-established indicator for LAMP reactions (Fischbach et al., 2015; Foo et al., 2020). Positive reactions can be identified by visible colour change and fluorescence under specific excitation. To evaluate this in our assay, we used calcein powder and MnCl_2_ from Sigma-Aldrich (St. Louis, MO, USA).

Since the fluorescence in this system depends on the calcein-Mn^2+^ interaction, different concentrations were tested. Additionally, as Mg^2+^ levels influence the signal, different Mg^2+^ concentrations were evaluated (Supplementary Table S2).

Here*, S. pneumoniae* detection was evaluated using bacterial suspensions of 10^8^, 10^6^, 10^4^, and 10^2^ CFU/mL, using the selected concentrations of Calcein (0.025 mm), Mn^2+^ (0.6 mM), and Mg^2+^ (8 mM) that provided optimal differentiation between positive and negative reactions.

SYTO dyes have been used to interpret LAMP results, with SYTO 9 noted for its low inhibitory effect and minimal product requirement for signal emission (Quyen et al., 2019). In this study, we used SYTO 9 nucleic acid green fluorescent stain, 5 mM in DMSO, from Invitrogen (Waltham, MA, USA). Tested concentrations included 10 μM, 5 μM, 0.625 μM, 0.50 μM, 0.75 μM, and 0.312 μM as reported by Quyen et al. (2019). DMSO was used as a diluent.

Finally, we tested the combination of HNB and SYTO 9. It has been reported that this combination can be used to distinguish negative and positive LAMP reactions (Li et al., 2023). Therefore, we evaluated different concentrations of these dyes in our assay. HNB was evaluated at 525 μM, 393.75 μM, 341.25 μM, 315 μM, 262.5 μM, 131.5 μM, and 78.75 μM, each combined with 0.25 μM, 0.5 μM, and 0.75 μM of SYTO 9.

For all dye selection experiments, a 0.5 McFarland suspension of *S. pneumoniae* strain ATCC 49619 and its specific primers was used as a positive control, after incubation at 99 °C for 10 min for nucleic acid extraction, and the LAMP reaction was performed at 65 °C for 60 min using a commercial thermoblock (Applied Biosystems, Thermal Cycler 2720). Fluorescence was visualized under blue light at 470 nm with a Safe Imager 2.0 transilluminator (Invitrogen, Waltham, MA, USA), and images were captured using a smartphone (Apple Inc., USA). Amplification was confirmed by electrophoresis on a 2.5% agarose gel run at 90 V for 30 min.

Once the optimal concentration of HNB (341.25 μM) and SYTO 9 (0.75 μM) was established, we assessed the effects of Mg^2+^ concentration and reaction temperature on the fluorescence observed at the end of the LAMP assay. Also, the stability of the fluorescence signal was evaluated over time.

For the evaluation of Mg^2+^ ions, concentrations of 6, 7, and 8 mM of Mg^2+^ were tested to assess their impact on fluorescence.

Temperature plays a key role in LAMP, as it influences the amount of product generated and, consequently, the end-point fluorescence. Therefore, LAMP reactions were performed at temperatures ranging from 60 to 65 °C. Optimal concentrations of HNB (341.25 μM), SYTO 9 (0.75 μM), and Mg^2+^ (7 mM), were used as determined in previous tests.

To assess the stability of the fluorescence obtained at the end of the LAMP reaction, using the optimal concentrations of HNB and SYTO 9 (Table 1), the LAMP reaction was performed using serial dilutions of *H. influenzae* ATCC 49766. Starting from a 0.5 McFarland suspension (approximately 1×10^8^ CFU/mL), 1:10 dilutions were prepared down to 10^2^ CFU/mL. Fluorescence was photographed at the end of the reaction (day 0) using a smartphone (Apple Inc., USA). Reaction tubes were stored protected from light, and an additional photo was taken at 52 days post-reaction.

**Table 1:** Final and optimal concentrations for the LAMP reaction.

Compound	Reagent	Reaction concentration	Volume (μL)
Isothermal amplification buffer	Tris–HCl	20 mM	2.5
	(NH_4_)2SO_4_	10 mM	
	KCl	50 mM	
	MgSO_4_	2 mM	
	Tween 20	0.1%	
MgSO_4_ solution 100 mM	MgSO_4_	4 mM	1
Betaine solution	Betaine	0.8 M	4
Deoxynucleotide (dNTP) Solution Mix (10 mM each)	dATP, dTTP, dGTP, dCTP	1.4 mM (each)	3.5
LAMP primers. 10X^a^	F3	0.2 μM	2.5
	B3	0.2 μM	
	FIP	0.8 μM	
	BIP	0.8 μM	
	LB	0.4 μM	
	LF	0.4 μM	
Bst 2.0 Warm Start	Bst Enzyme	8 U	1
HNB at 8.53125 mM	HNB	341.25 μM	1
SYTO 9 at 18.75 μM	SYTO 9	0.75 μM	1
Sample^b^	Target DNA	–	8.5
Total reaction volume	25 μL		
Seal of mineral oil^c^	15 μL		

a In the detection of *M. pneumoniae*, the primer LB was not considered.

b To prevent contamination.

c Bacterial suspensions or genomic DNA in the case of *M. pneumoniae*.

### 2.2 Standardisation of the detection panel of the main pneumonia-causing bacteria in children

Once we choose the use of HNB and SYTO 9 as proper dyes, we set up the LAMP reaction for the detection of the pathogens included in the panel. Those were *S. pneumoniae*, *S. aureus, H. influenzae*, *K. pneumoniae*, and *M. pneumoniae*. The primers used for each bacterium are listed in Table 2 along with the corresponding references from which they were obtained where they were taken.

**Table 2:** Primer sets used for the detection of *S. pneumoniae*, *S. aureus*, *H. influenzae and M. pneumoniae.*

Target bacterium	Primer	Secuencia 5′-3′	Target gene	Reference
*Streptococcus pneumoniae*	F3	CTGGAGGAAGCACACAGA	*lyt*A	Kang et al. (2012)
	B3	GTCTGGTTTGAGGTAGTACC		
	FIP	CACCTTCTTCGTTGAAATAGTACCA-CTGGTTCGACAACTCAGG		
	BIP	GACAGGCTGGGTCAAGTACAA-TGGATAAAGGCATTTGATACC		
	LF	AGCGATTTTCTTCCAGCC		
	LB	CTTAGACGCTAAAGAAGGCG		
*Staphylococcus aureus*	F3	TGAATCATGATGGCGAGAT	*fem*A	Kang et al. (2012)
	B3	CGTGTTTCTTTTTCTAAGTCCA		
	FIP	ATGGAATCCAGTATGTTCAAATCCTAGGTAATGCTGGTAATGATTGG		
	BIP	AAGGATTTGATCCTGTGCTACAAATTTAATGATGTCATCTGCTGTT		
	LF	AAGTTACTCATTTTATCAAAGA		
	LB	TTCGTTATCACTCAGTGTTAGA		
*Haemophilus influenzae*	F3	GCAGATGCAGTTAAAGGTT	*Omp*P6	Kang et al. (2012)
	B3	GCTAATTGGTTAAATTACAAACGA		
	FIP	ACCTAATACTGCAGGTTTTTCTTCA-GGTAAAGGTGTTGATGCTGG		
	BIP	GAAGCTGCATATTCTAAAAACCGTC-AAAAATGGATCCTGTTTTTCAAGT		
	LF	CCGTAAGATACTGTGCCTAATT		
	LB	GCAGTGTTAGCGTACTAATTCT		
*Mycoplasmoides pneumoniae*	F3	CCACCTAGTGATTTGGAAGA	CARDS toxin gene	Petrone et al. (2015)
	B3	GGACAAAGAAGATTTTCGAAGTT		
	FIP	GCTGAACATCAACAAAGAAGGTGCATTGTTGATGAATGTACTACCCA		
	BIP	ATACCCCACAATTAAGTGGTTGATTCATAGAATATCTGTCCATCTGG		
	LF	CTGCACGCATAGTAACAAACTG		

In the case of *K. pneumoniae* detection, primers previously reported were initially tested, but did not perform adequately in our reaction conditions (data not shown). Therefore, a new primer set was designed. For this purpose, 502 sequences of the *K. pneumoniae* haemolysin *khe* gene were downloaded from the NCBI GenBank database, and aligned them using the MAFFT tool of UGene Ver. 52.1 (Okonechnikov et al., 2012), and identified conserved regions using WebLogo ver. 3.7.9 (Crooks et al., 2004). The primer set was designed using PrimerExplorer V5, from http://primerexplorer.jp/lampv5e/index.html (Eiken Chemical Co., Ltd., Tokyo, Japan).

The specificity of the designed primer set was analysed *in silico* using the BLASTn tool from the National Center for Biotechnology Information (NCBI) available on https://blast.ncbi.nlm.nih.gov/blast/Blast.cgi?PROGRAM=blastn&PAGE_TYPE=BlastSearch&LINK_LOC=blasthome.

Subsequently, *in vitro* LAMP assays were conducted to assess cross-reactivity. Initially, tests included panel bacteria: *S. pneumoniae* ATCC 49619, *S. aureus* ATCC 25923, *H. influenzae* ATCC 49766, and purified DNA *from M. pneumoniae* (ATCC 29342DQ). Additional assessments included: *S. aureus* ATCC 29213, *Escherichia coli* NCTC 13846, *E. coli* ATCC 25922, *Acinetobacter baumannii* ATCC 17978, *A. baumannii* ATCC 19606, *Proteus mirabilis*, *Aeromonas hydrophila*, *Pseudomonas aeruginosa*, *Acinetobacter nosocomialis*, *A. junii*, *A. pittii*, *Salmonella Enterica*, *Serratia marcensens*, *Enterobacter cloacae*, *E. asburiae*, and *K.* var*iicola*. These last 11 strains were clinical isolates.

For this, DNA was extracted from each strain by heating 0.5 MacFarland’s suspension at 99 °C for 10 min, and LAMP reactions followed the conditions described in Table 1, using *K. pneumoniae* ATCC 13883 (0.5 McFarland) as a positive control. Amplification was confirmed by 2.5% agarose gel electrophoresis at 90 V for 30 min.

#### 2.2.1 Analytical sensitivity assessment for each target bacterium

*Streptococcus pneumoniae* ATCC 49619, *S. aureus* ATCC 25923, *H. influenzae* ATCC 49766, and *K. pneumoniae* ATCC 13883 were used to determine the limit of detection (LoD) of the proposed LAMP reaction. For each strain, six serial 1:10 dilutions were done in sterile saline solution, starting from a 0.5 McFarland bacterial suspension (approximately 1.5 ×10^8^ CFU/mL).

To precisely determine the LoD in CFU/mL, 100 μL of the previous bacterial suspensions were cultured on 5% blood agar for *S. pneumoniae*, *S. aureus*, and *K. pneumoniae*. Cholate agar was used for *H. influenzae*. Plates were incubated for 24 h at 37 °C (for *H. influenzae,* plates were incubated in a CO_2_ atmosphere). After incubation, colonies were counted, and the CFU/mL were calculated accordingly.

After culturing, dilutions were subsequently boiled at 99 °C for 10 min to extract DNA. LAMP reactions were performed at 65 °C for 60 min, followed by exposure of the reaction tubes to blue light (470 nm) using Invitrogen’s Safe Imager 2.0 transilluminator (Waltham, MA, USA). Photos were taken using a smartphone (Apple Inc., USA).

In the case of *M. pneumoniae,* Quantitative Genomic DNA from *Mycoplasmoides pneumoniae* strain M129-B7 (ATCC 29342DQ) purchased from ATCC (Manassas, VA, USA) was used based on the manufacturer’s reported concentration of 3.0 ×10^5^ genome copies/μL. Starting from this concentration, serial 1:10 dilutions were performed down to 1.27 ×10^2^ genome copies/reaction to determine the minimum detectable concentration.

To confirm amplification, 2.5% agarose gel electrophoresis was performed at 90 V for 30 min for each LAMP reaction.

#### 2.2.2 Effect of bacterial load on time to positivity

This analysis aimed to assess fluorescence changes during the standardised LAMP reaction, and to determine the time required for a signal emergence in positive reactions, focusing on panel bacteria such as *S. pneumoniae*, *S. aureus*, and *H. influenzae,* which may act as colonizers rather than true pathogens.

Bacterial suspensions at McFarland standards 4, 2, 1, and 0.5 were prepared. From the 0.5 suspension (approximately 10^8^ CFU/mL), 1:10 serial dilutions were made down to 10^3^ CFU/mL. DNA was extracted as previously described (99 °C for 10 min) for use in the LAMP reaction.

The reaction was monitored every 5 min, over a total period of 1 h, with all assays performed in triplicate. For this, 13 reaction tubes were prepared from each bacterial suspension tested, one for each 5-min interval and one negative control. Reactions were conducted at 65 °C, and at each interval, a tube was removed and placed on ice to stop the amplification. After 60 min, all tubes were exposed to blue light to visualise fluorescent signals.

### 2.3 Proof-of-concept testing with clinical samples

To verify that the standardised LAMP reaction performed adequately in a clinical context, 25 respiratory samples from patients with respiratory tract infections were analysed. These included samples positive for each bacterium in the panel as well as negative samples. These samples included nasopharyngeal aspirates (NAS), bronchoaspirates (BAS), endotracheal aspirates (EAS), sputum, and bronchoalveolar lavages (BAL) collected at Hospital Clinic of Barcelona and Hospital Sant Joan XXIII in Tarragona, Spain. All samples were residual material from the routine diagnostic workflow; therefore, microbiological identification was available for each sample. Identification was performed by bacterial culture or by PCR in the case of samples positive for *M. pneumoniae*.

For the LAMP reaction, 25 μL of the raw sample was mixed with 500 μL of Milli-Q water to reduce viscosity and cellular load. A simple nucleic acid extraction was then performed by heating the diluted sample at 99 °C for 10 min. Subsequently, 8.5 μL of this preparation was used as a template in the LAMP reaction, using the components described in Table 1. For each bacterium included in the detection panel, a specific reaction mix containing its corresponding primers was prepared.

To ensure proper reaction performance and exclude the possibility of sample inhibition, an inhibition control (IC) was included in each assay. The IC contained 5 μL of sample extract, 3.5 μL of a *S. pneumoniae* ATCCC 49619 suspension at 1.5 ×10^8^ CFU/mL, and specific primers for this bacterium. The IC was required to always produce a positive result to validate each assay.

Reaction temperature and time were maintained as described in previous assays (65 °C, 60 min). End-point fluorescence was assessed by exposing the reaction tubes to blue light and imaging the signal with a smartphone camera.

Ethics statement: This study was conducted with leftover clinical samples and was approved by the Ethics Committee of Hospital Clinic of Barcelona. Registration No. HCB/2023/0652.

## 3 Results

### 3.1 The use of SYBR safe did not allow a visual interpretation of the LAMP reaction

No clear distinction between negative and positive reactions was observed with any of the SYBR Safe concentrations tested (Supplementary Figure S1). Higher concentrations resulted in a stronger fluorescent signal visible to the naked eye, but also in negative controls. Additionally, electrophoresis showed that increased SYBR Safe concentrations correlated with reduced reaction product.

Although slight differences in fluorescence intensity were noted at 3.2X, 16X, and 1X, they were insufficient for reliable visual interpretation of the reaction results.

### 3.2 The use of calcein-Mn^2+^ did not provide a clear differentiation between positive and negative LAMP reactions and could affect the reaction efficiency

Calcein-Mn^2+^ concentrations were chosen based on previous studies (Fischbach et al., 2015; Petrone et al., 2015; Foo et al., 2020) commonly using 0.05 mM calcein. When this was combined with 0.5, 1.4, 1.6, and 1.8 mM of Mn^2+^, no clear distinction between positive and negative tubes was observed. However, at 0.025 mM calcein and 0.5 mM of Mn^2+^, visible colour differences and fluorescence signals between positive and negative reactions were evident at the end of the LAMP reaction (Supplementary Figure S2).

Therefore, calcein concentrations of 0.025 mM and Mn^2+^ ranging from 0.5 to 0.9 mM were tested. Slight colour and fluorescence differences between positive and negative reactions were visible to the naked eye. However, a background fluorescence signal persisted in negative tubes. Using 0.025 mM calcein and 0.6 mM Mn^2+^, only high bacterial loads of *S. pneumoniae* (above 10^6^ CFU/mL) showed a clear distinction.

To improve differentiation between fluorescent in negative and positive tubes, the Mg^2+^ concentration was adjusted due to its influence on fluorescence intensity. With calcein fixed at 0.025 mM, and Mg^2+^ at 5.5 mM, varying Mn^2+^ from 0.5 to 1.375 mM reduced background signal in negative tubes (Supplementary Figure S2). However, this also reduced fluorescence intensity and the amount of reaction products, compromising the efficiency of the reaction and making result interpretation more difficult.

### 3.3 If only SYTO 9 is used, it is not visually possible to distinguish between positive and negative LAMP reactions

Although slight differences were observed between positive and negative reactions at various SYTO 9 concentrations, this dye alone was insufficient for reliable visual differentiation. As SYTO 9 concentration increased, fluorescent intensity in positive tubes also increased; however, a background signal remained in negative tubes, similar to previous tested indicators.

While some variations were visible to the naked eye, particularly at concentrations above 0.5 μM of SYTO 9 (Supplementary Figure S3), these were not distinct enough for accurate interpretation, rendering SYTO 9 unsuitable as a standalone dye.

### 3.4 The use of SYTO 9 combined with HNB is an adequate method to distinguish between negative and positive LAMP reactions

#### 3.4.1 Search for the concentrations that provide optimal contrast between positive and negative LAMP reactions

**Figure 1:**
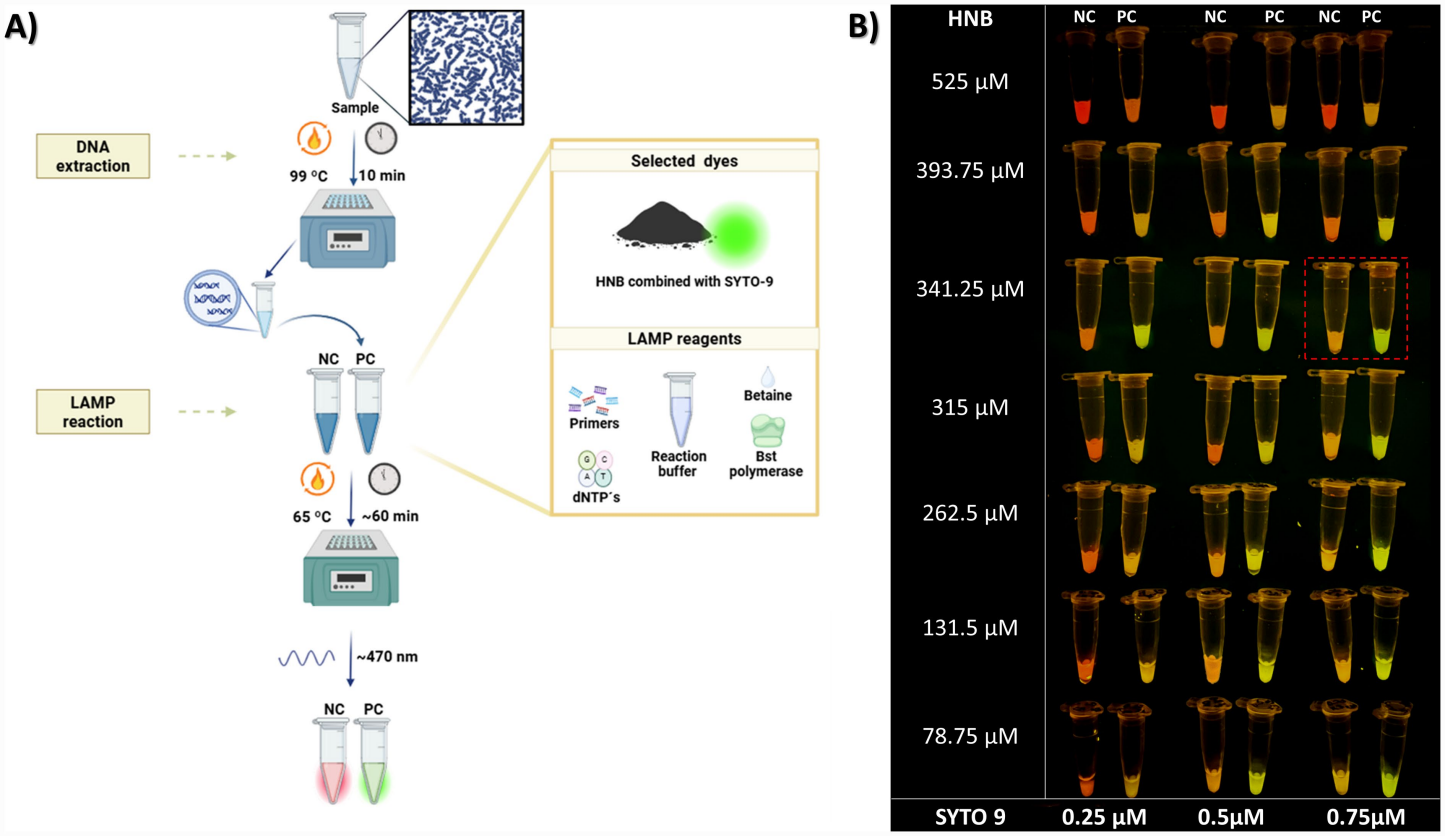
The use of HNB and SYTO 9 allow clear differentiation between positive and negative LAMP reactions. LAMP protocol for detecting of the main pneumonia-causing bacteria in children **(A)**. The workflow was divided into two phases: DNA extraction and the LAMP reaction, using HNB and SYTO-9 as dyes. Positive reaction tubes showed green fluorescence, while negative tubes displayed a reddish-orange signal when exposed to blue light (470 nm). Different tested concentrations of HNB and SYTO 9 in the LAMP reaction **(B)**. Among the concentrations tested, 341.35 μM HNB and 0.75 μM SYTO 9 were selected as they provided a clear contrast between positive and negative reactions. Although higher SYTO 9 levels increased fluorescence in positive samples, increasing HNB concentrations made both positive and negative tubes appear more reddish, thereby reducing visual distinction.

The combination of SYTO 9 and HNB improved visual distinction between positive and negative LAMP results. HNB indicated negatives via a colour change to a reddish signal, while SYTO 9 green fluorescence marks positives, simplifying interpretation by eye. Across the tested concentrations, variations in fluorescent intensity and colour were observed at the end of the LAMP reaction. Higher HNB concentrations intensified red tones in both negative and positive tubes, masking SYTO 9’s green fluorescence and producing an orange hue in positive tubes. Conversely, reducing HNB concentration and increasing SYTO 9 allowed its green signal to dominate, resulting in orange or yellow colours in negative tubes (Figure 1).

At first glance, the most noticeable contrast was achieved with 0.75 μM SYTO 9 and 341.25 μM HNB.

#### 3.4.2 Mg^2+^ variations in the reaction affect the fluorescent signal emitted by HNB

**Figure 2:**
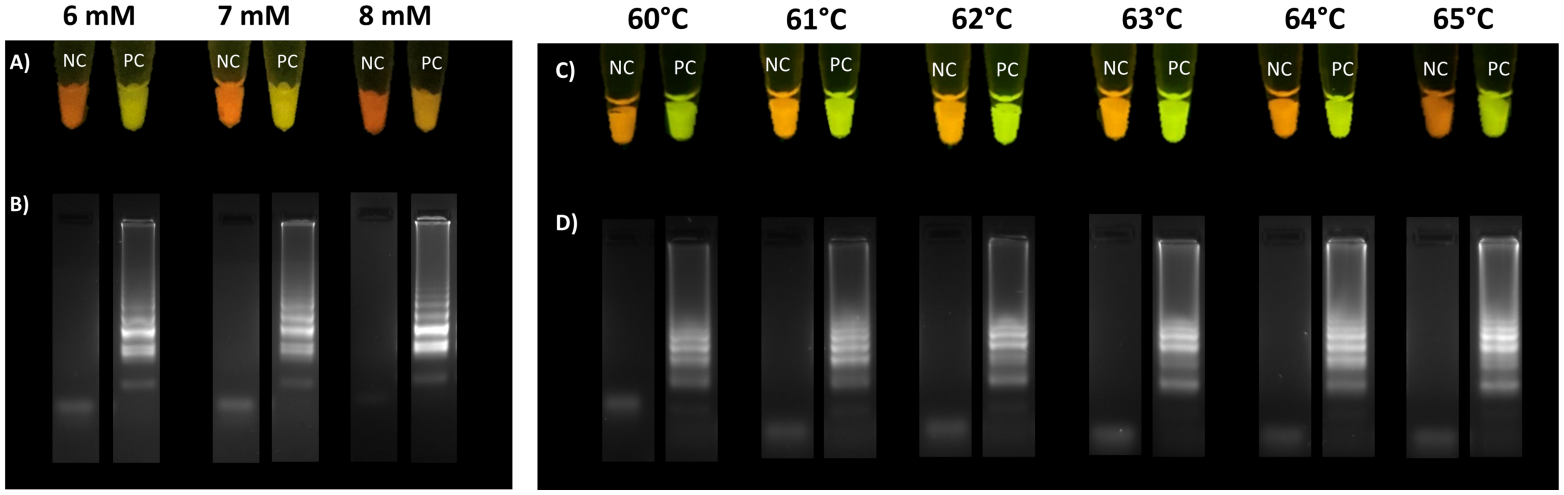
Effect of Mg^2+^ concentration and temperature variations on the LAMP reaction. Increasing Mg^2+^ concentration intensifies the reddish fluorescence signal emitted by HNB, resulting in a deeper colour in negative tubes **(A)**, alongside producing a higher number of products **(B)**. While increasing the reaction temperature does not have an influence on the fluorescent signal observed in both positive and negative tubes **(C)**, this temperature increase generates a higher amount of reaction products **(D)**.

As Mg^2+^ concentration increases in the LAMP reaction, the red fluorescence from HNB intensifies, improving visual discrimination of negative reactions. However, in positive reactions, SYTO 9’s green signal is also affected, appearing orange at 8 mM Mg^2+^ (Figure 2).

Since Mg^2+^ is essential for polymerase activity, 6 mM was selected as the optimal concentration to ensure amplification without compromising the interpretation of results based on the observed fluorescent signals.

#### 3.4.3 Increasing the reaction temperature did not affect the fluorescent signal emitted by SYTO 9 and HNB

Temperatures between 60 and 65 °C had little effect on fluorescence in positive and negative tubes. However, more product was generated as the temperature increased (Figure 2), making 65 °C the optimal temperature for the LAMP reaction.

#### 3.4.4 With the use of SYTO 9 combined with HNB, the fluorescent signal emitted remains for weeks

The fluorescent signals from HNB and SYTO 9 remained stable, allowing distinction between positive and negative reactions even after 52 days.

Over time, negative tubes appeared brighter red-orange while positive signals faded to yellow (Supplementary Figure S4).

### 3.5 Detection panel of the main pneumonia-causing bacteria in children

#### 3.5.1 LoD of *Streptococcus pneumoniae*, *Staphylococcus aureus*, *Haemophilus influenzae*, and *Mycoplasmoides pneumoniae*

The LoD was defined as the lowest bacterial dilution showing a visible positive signal after LAMP and confirmed by colony counts on agar plates.

**Figure 3:**
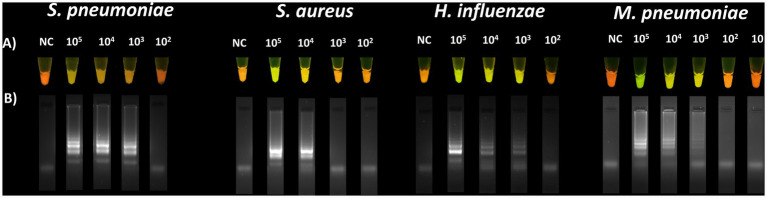
Sensitivity of the LAMP reaction for the detection of *S. pneumoniae*, *S. aureus*, *H. influenzae*, and *M. pneumoniae.* Visual LoD determined for the detection of *S. pneumoniae* (3.9 ×10^3^ CFU/mL), *S. aureus* (1.7 ×10^5^ CFU/mL), *H. influenzae* (8.2 ×10^3^ CFU/mL), and *M. pneumoniae* (1.27 ×10^3^ genome copies/reaction) **(A)**. Verification of amplification by 2% agarose gel electrophoresis **(B)**.

The determined LoD for *S. pneumoniae* was 3.9 ×10^3^ CFU/mL, 1.7 ×10^5^ CFU/mL for *S. aureus*, 8.2 ×10^3^ CFU/mL for *H. influenzae,* and 1.27 ×10^3^ genome copies/reaction for *M. pneumoniae* (Figure 3).

#### 3.5.2 Primer design for the detection of *Klebsiella pneumoniae*

Alignment of sequences of the *khe* gene showed high conservation, with minor variations at positions 228 (G and A), 366 (C and T), and 471 (T and C), all at a single base (Supplementary Figure S5).

All the bases were considered in designing the Kpne-AMT primer set, which includes F3, B3, FIP, BIP, LF, and LB primers (Table 3). These primers bind to regions of the *khe* sequence as shown in the Supplementary Figure S6.

**Table 3:** Designed Kpne-AMT primers for *K. pneumoniae* detection.

Primer	Sequence 3′-5′	Gen sequence position 5′-3′	Length
Kpne-AMT-F3	ACGGCTATCTCTGGAAGCT	242	19
Kpne-AMT-B3	GCTTACCGTCGTGTGGAC	458	18
Kpne-AMT-FIP	GACGAACTTCCTGCTCGGTGTT-TGGGTTTTCCCGCTGGTA	278–29; 322–343	22 + 18
Kpne-AMT-BIP	ATTACCCGCTCAATCCCGGC-GAAGAACTGCGGCGGATG	439–456; 386–405	20 + 18
Kpne-AMT-LB	TGAGAAAGGTGTGGCAGATGC	299–319	21
Kpne-AMT-LF	ACGCGCCAGGATCGTT	415–430	16

BLASTn analysis showed no significant similarity of the designed primers with species outside the *K. pneumoniae* complex. *In vitro* test confirmed no cross-reactivity with panel bacteria or other Gram-positive and Gram-negative species (Supplementary Figure S7), demonstrating high specificity.

**Figure 4:**
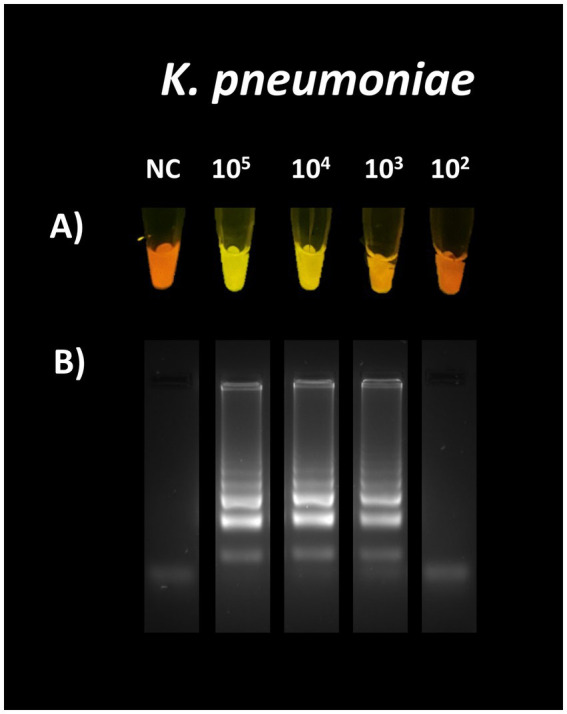
Sensitivity of the designed primers for the detection of *K. pneumoniae.* Visual LoD **(A)**. The calculated LoD was 1.5 ×10^4^ CFU/mL. Amplification was confirmed by 2% agarose gel electrophoresis **(B)**.

A positive signal was observed to the naked eye at 1.5 ×10^4^ CFU/mL, establishing the LoD (Figure 4).

### 3.6 Bacterial load effect on the detection time of potentially colonizing bacteria

For bacteria acting as colonisers rather than true pathogens, lower bacterial loads led to longer detection times for positive fluorescence. In *S. pneumoniae*, positive signals appeared between 30 and 35 min after the reaction started in high bacterial loads (RANGE), while detection times beyond 55 min may indicate low bacterial load concentrations (≥10^7^ CFU/mL).

For *S. aureus*, a similar pattern was observed: high concentrations (≥10^7^ CFU/mL) showed positivity from 30 min onwards, while lower bacterial loads (≤10^4^ CFU/mL) extended detection times with positive signals appearing at 55 min or after. Longer detection times correlate with lower bacterial concentrations.

**Figure 5:**
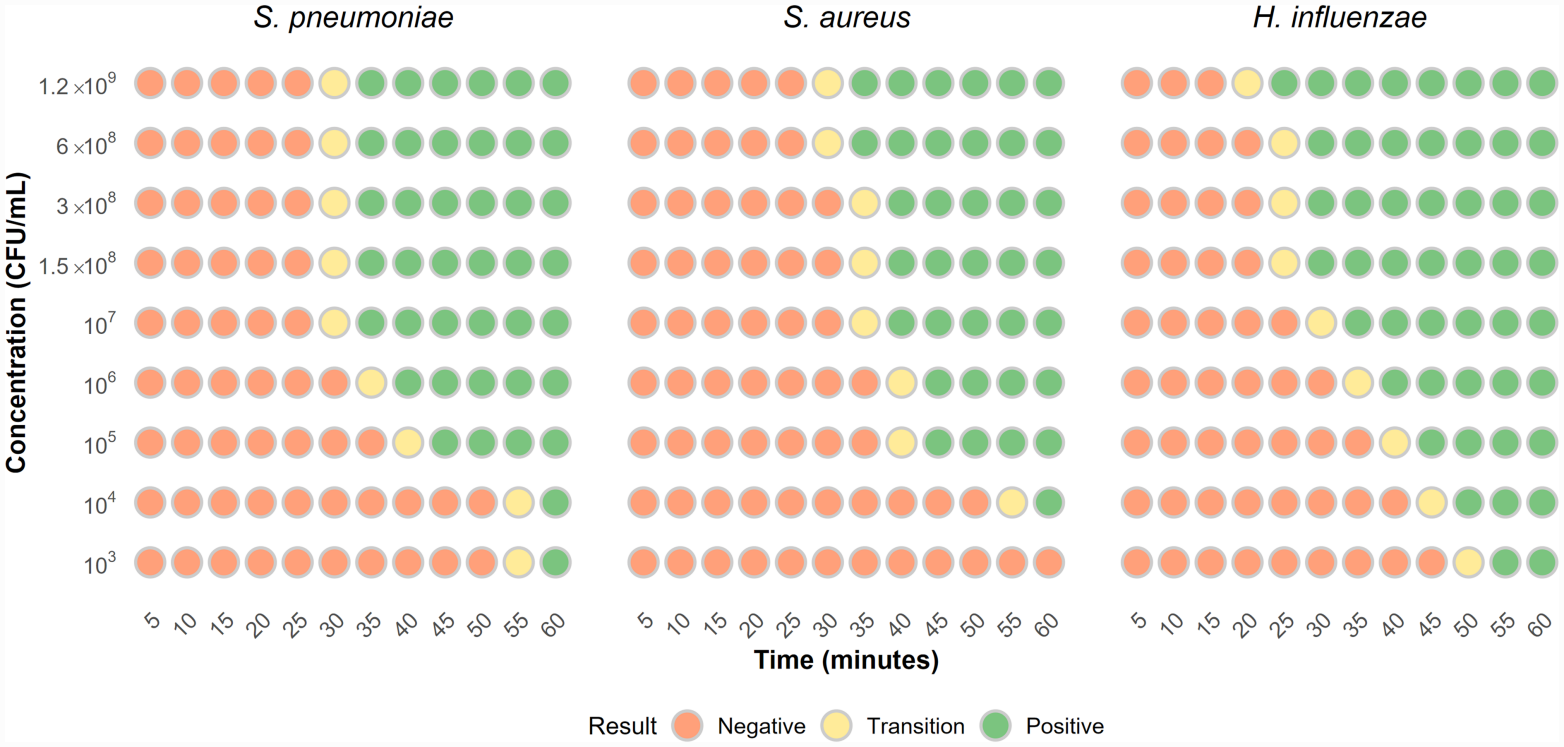
Relationship between time to positivity and bacterial load for LAMP detection of *S. pneumoniae*, *S. aureus*, and *H. influenzae*. As the bacterial concentration decreased, the time required for detection increased across all three pathogens. For *S. pneumoniae*, high concentrations produced a clear positive result within 35 min. However, concentrations near the LoD (10^4^ and 10^3^ CFU/mL) required more than 55 min to be considered positive. Similarly, *S. aureus* showed a positive signal at 35 min when tested at high concentrations, whereas lower concentrations (around 10^4^ CFU/mL) required over 45. In the case of *H. influenzae*, the highest concentration yielded a visible positive reaction at 25 min, while lower concentrations needed up to 45 min. Transition phase: In all cases, there was a stage where tubes began to show a greenish signal, indicating the onset of positivity, although the color had not yet fully developed to a strong green signal.

In *H. influenzae*, positive signals started to appear between 20 and 25 min at high bacterial concentrations (≥10^7^ CFU/mL), and after 45 min when the load was low (≤10^4^ CFU/mL) (Figure 5).

### 3.7 The proposed LAMP technique performs reliably with respiratory clinical samples

The performance of the standardised reaction was evaluated using clinical samples. The assay was successfully performed and could be accurately read despite inherent sample characteristics such as viscosity or traces of blood inclusion.

As shown in Table 4, all target bacteria were correctly identified by LAMP, consistent with culture or PCR results (in the case of *M. pneumoniae*). In some samples, additional bacteria were detected: 5-STD, where both *S. pneumoniae* and *H. influenzae* were identified, and in sample 8-STD, both *M. pneumoniae* and *S. pneumoniae*. All five negative samples remained negative. Fluorescent signals are shown in Supplementary Figure S8.

**Table 4:** Evaluation of the standardised LAMP reaction using clinical samples positive and negative for the target bacteria.

Sample ID	Sample type	Culture result	Reported bacterial load (UFC/mL)	LAMP result
*Streptococcus pneumoniae*				
4-STD	Nasopharyngeal aspirate	*S. pneumoniae*	NA	*S. pneumoniae*
5-STD	Nasopharyngeal aspirate	*S. pneumoniae*	NA	*S. pneumoniae/H. influenzae*
13-STD	Bronchoaspirate	*S. pneumoniae/H. influenzae*	*H. influenzae*: 200,000 *S. pneumoniae*: 40,000	*S. pneumoniae/H. influenzae*
37-STD	Nasopharyngeal aspirate	*S. pneumoniae*	NA	*S. pneumoniae*
*Staphylococcus aureus*				
2-STD	Endotracheal aspirate	*S. aureus*	>100,000	*S. aureus*
33-STD	Endotracheal aspirate	*S. aureus*	40,000	*S. aureus*
40-STD	Endotracheal aspirate	*S. aureus*	500,000	*S. aureus*
49-STD	Sputum	*S. aureus*	NA	*S. aureus*
67-STD	Endotracheal aspirate	*S. aureus*	ND	*S. aureus*
72-STD	Endotracheal aspirate	*S. aureus*	>1,000,000	*S. aureus*
*Haemophilus influenzae*				
13-STD	Bronchoaspirate	*S. pneumoniae/H. influenzae*	*H. influenzae*: 200,000 *S. pneumoniae*: 40,000	*S. pneumoniae/H. influenzae*
16-STD	Nasopharyngeal aspirate	*S. pneumoniae/H. influenzae*	NA	*S. pneumoniae/H. influenzae*
19-STD	Nasopharyngeal aspirate	*H. influenzae*	NA	*H. influenzae*
*Klebsiella pneumoniae*				
43-STD	Endotracheal aspirate	*K. pneumoniae/S. maltophilia*	>2000,000	*K. pneumoniae*
64-STD	Bronchoaspirate	*K. pneumoniae*	400,000	*K. pneumoniae*
79-STD	Endotracheal aspirate	*K. pneumoniae*	400,000	*K. pneumoniae*
83-STD	Bronchoaspirate	*K. pneumoniae*	>2000,000	*K. pneumoniae*
91-STD	Bronchoaspirate	*K. pneumoniae*	100,000	*K. pneumoniae*
Mycoplasmoides pneumoniae*				
7-STD	Nasopharyngeal aspirate	*S. pneumoniae/M. pneumoniae*	NA	*M. pneumoniae/S. pneumoniae*
8-STD	Nasopharyngeal aspirate	*M. pneumoniae*	NA	*M. pneumoniae/S. pneumoniae*
11-STD	Nasopharyngeal aspirate	*M. pneumoniae/S. aureus*	NA	*M. pneumoniae/S. aureus*
*Negatives*				
23-STD	Bronchoalveolar lavage	Negative	NA	Negative
47-STD	Endotracheal aspirate	Negative	NA	Negative
54-STD	Endotracheal aspirate	Negative	NA	Negative
62-STD	Endotracheal aspirate	Negative	NA	Negative
66-STD	Endotracheal aspirate	Negative	NA	Negative

NA, not applicable; ND, not determined.

*Mycoplasmoides pneumoniae identification was done by PCR.

## 4 Discussion

LAMP technology is widely used for pathogen detection due to its high sensitivity, specificity, simplicity, and rapid results (Atceken et al., 2023) meeting WHO criteria for tuberculosis diagnosis (WHO, 2016). Although it has been extensively applied to viral respiratory pathogens, especially after the SARS-CoV-2 pandemic, few studies focus on key bacterial agents of childhood pneumonia (Pneumonia in children: What you need to know | UNICEF, 2024). Many existing methods rely on complex detection systems, limiting use in high-incidence, low-resource settings.

This study describes a LAMP-based panel designed to detect key bacterial causes of childhood pneumonia, aiming to standardise a reliable, user-friendly method with straightforward interpretation. As a first step, we tested various dyes to enable naked-eye interpretation of LAMP results without specialised equipment.

SYBR Safe, an accessible dye that intercalates into dsDNA and fluoresces when excited at 280 nm or 502 nm, was initially evaluated (Kolbeck et al., 2021). This was demonstrated in the results, where positive reactions showed stronger fluorescence than negatives, but residual signals in negative tubes made interpretation difficult. This background may stem from SYBR Safe binding not only to dsDNA, but also to primers and single-stranded DNA (ssDNA), as reported for SYBR Green (Shaik et al., 2008; Dragan et al., 2012). Higher dye concentrations also reduced efficiency and product yield, leading us to discard them. Though SYBR Safe has been used in LAMP for *Leishmania* detection (Thita et al., 2019) visual interpretation remained challenging. Later, combining with gold nanoparticles improved clarity, but only in post-reaction steps (Ruang-areerate et al., 2021).

Since SYBR Safe lacked a clear visual distinction, we switched to calcein chelated with Mn^2+^. Calcein’s fluorescence is quenched by Mn^2+^, but during LAMP amplification, generated pyrophosphate binds and precipitates Mn^2+^, releasing fluorescence when excited near 480 nm (Mansour et al., 2015). Calcein also enables colour changes, slightly yellow for negative and green for positive (Tomita et al., 2008). Optimizing calcein-Mn^2+^ and Mg^2+^ concentrations is crucial to visually distinguishing LAMP results. In our study, adjustments had little impact on fluorescence or colour change. A persistent background signal in all tubes, including negatives, made interpretation difficult and risked false positives. Sensitivity was also low, with only high concentrations of *S. pneumoniae* showing a clear positive signal. This may stem from incomplete calcein-Mn^2+^ chelation, leaving residual fluorescence. Increasing Mn^2+^ reduces this background but also impairs reaction efficiency, likely due to Mn^2+^ competing with Mg^2+^, which the polymerase requires. While some polymerases can use Mn^2+^ as a cofactor, it often reduces performance and may destabilise important LAMP structures like loops or hairpins (Millonig et al., 2009; Vashishtha and Konigsberg, 2016).

Adjusting the [Mn^2+^: Mg^2+^] ratio can enhance contrast between positive and negative LAMP reactions. However, in our system, even at different ratios, the distinction remained unclear. Although a 4:1 ratio has been reported to improve visual differentiation (Petrone et al., 2015), residual fluorescence persisted in negative tubes, making result interpretation difficult.

We next evaluated SYTO 9, a dye that binds to dsDNA and emits strong green fluorescence when excited at 480–500 nm (Monis et al., 2005). Compared to SYBR Safe dyes, SYTO 9 offers less inhibition, stronger fluorescence, and a better signal-to-noise ratio, reducing false positives (Ma et al., 2019). However, when used alone, it failed to clearly differentiate positives from negatives by eye, as both emitted similar fluorescence. Although SYTO dyes have been used in LAMP, those studies typically relied on real-time systems with sensitive detectors to capture subtle differences (Chen and Ge, 2010; Patel et al., 2013; Oscorbin et al., 2016; Quyen et al., 2019). Fluorescence in negative tubes may result from SYTO 9 binding to primers or template DNA, even without amplification (Meagher et al., 2018). To address this, a dual-dye system was proposed, combining SYTO 9 to mark positives and a second dye to mask its background signal. SYTO 9 was paired with HNB as previously reported (Li et al., 2023). HNB emits reddish-orange fluorescence when interacting with Mg^2+^ under ~470 nm light. In positives, SYTO 9 binds to amplified dsDNA, emitting a strong green signal. In negatives, HNB masks any weak SYTO 9 fluorescence, offering a clear visual contrast: green for positives, reddish-orange for negatives.

We adopted this dye combination with modifications to optimise concentrations for our LAMP. HNB fluorescence was initially too faint to allow a clear visual distinction between positive and negative reactions. The optimal concentrations were 341.25 μM for HNB, 13 times higher than previously reported, and 0.75 μM for SYTO 9. Higher HNB intensified the red hue in negatives but gave positives a reddish-orange tone. Increasing SYTO 9 countered this, especially at low HNB levels, highlighting the need to fine-tune dye concentrations for each LAMP system.

Mg^2+^ concentration influences both enzyme activity and fluorescence intensity (Oscorbin and Filipenko, 2023), due to its interaction with HNB, which produces the reddish signal in negative tubes. We tested Mg^2+^ concentrations between 6 and 8 mM, assessing fluorescence and product yield. Higher Mg^2+^ enhanced amplifications but also strengthened HNB fluorescence, masking SYTO 9’s positive green signal and shifting colour to orange. Since Mg^2+^ affects HNB’s absorptions and emission spectra (Goto et al., 2009; Ding et al., 2015) we chose a 6 mM Mg^2+^. It provided clear green fluorescence in positives, a distinct red in negatives, and strong amplification.

Reaction temperature affects enzyme performance. At 65 °C, LAMP produced the most intense band pattern, aligning with its reported optimum (Oscorbin and Filipenko, 2023). Components like KCl, H_3_PO_4_, and Tris–HCl can dampen HNB’s reddish fluorescence, but this effect is less noticeable above 63 °C, even after cooling to 4 °C (Li et al., 2023). Running the reaction at 65 °C helps maintain a strong, clear HNB signal, supporting effective visual interpretation.

Both SYTO 9’s green fluorescence and HNB’s reddish signal remained stable for over 52 days, far longer than the 9 days previously reported (Li et al., 2023). Using higher dye concentrations and storing samples away from light likely preserved signal clarity, allowing greater flexibility for reading or checking results after amplification. Over time, SYTO 9’s green fluorescence faded to greenish-yellow. Although photobleaching was limited by dark storage, reversible binding to amplification products may also explain this event (Biebricher et al., 2015; Stiefel et al., 2015). In contrast, HNB fluorescence intensified, possibly due to stronger interactions with Mg^2+^ developing over time (Goto et al., 2009).

Under the established reaction conditions, the sensitivity assay showed a LoD of 3.9 ×10^3^ CFU/mL for *S. pneumoniae*, matching previous study using the same primers, which reported 10^3^ copies/mL (Kang et al., 2012). This aligns with other reports with LoD values of 10^3^ CFU/mL (de Paz et al., 2020; Si et al., 2021) using real time detection systems are usually more sensitive than the visual method. These results suggest the method’s suitability for use in low-resource settings.

The LoD for *S. aureus* was 1.7 ×10^5^ CFU/mL, aligning with a previous study using the same primers (10^5^ copies/mL) (Kang et al., 2012) and others that have reported between 10^4^ (Si et al., 2021) and 10^5^ CFU/mL by flanking the same gene (*fem*A) (Vergara et al., 2020; Kim et al., 2022). For *H. influenzae,* our LoD of 8.2 ×10^3^ CFU/mL was slightly lower than the 10^5^ copies/mL reported (Kang et al., 2012). This difference likely reflects methodological variations, as our assay used bacterial suspension at known concentrations rather than clinical cut-offs. Overall, our findings are consistent with reported LoDs of 10^3^ CFU/mL (Kim et al., 2011; Takano et al., 2017; Wang et al., 2022).

As shown in Table 4, a clinical sample positive for *S. aureus* with a bacterial load of 4 ×10^4^ CFU/mL was successfully detected by LAMP, despite being below the reported LoD. This may reflect differences between controlled suspensions and clinical matrices, which can affect DNA availability; for instance, some bacteria in clinical samples may be lysed, releasing DNA and facilitating detection (Ren et al., 2020). Additionally, the LoD is an estimated threshold, and stochastic amplification can occasionally yield positive results below this value.

In *M. pneumoniae*, the LoD detected in our study (1.27 ×10^3^ genome copies/reaction) was higher than previously reported (Petrone et al., 2015) likely due to differences in the readout of the reaction. While droplet digital PCR (ddPCR) can detect as few as 2.9 copies/reaction of *M. pneumoniae* (Zhao et al., 2023) LAMP is simpler and easier to perform. Given that clinical loads above 10^5^ copies/mL indicate active infection, the sensitivity achieved with the standardised LAMP assay remains appropriate for detecting this bacterium (Liu et al., 2019; Shi et al., 2019; Zhao et al., 2020).

An initial primer set for *K. pneumoniae* described previously failed to produce adequate amplification under our reaction conditions (data not shown). Therefore, a new set was designed targeting the *khe* gene encoding haemolysin. Alignment of 502 nt sequences of this gene showed high conservation, with only three variations, increasing the likelihood of detecting clinically relevant strains. *In silico* analysis showed high specificity to *K. pneumoniae* complex, and through *in vitro* analysis, no cross-reactivity was detected with other bacteria, including closely related species, confirming the analytical specificity and reducing the risk of false positives (Yin-Ching et al., 2002; Feng et al., 2023). This confirms the specificity and reliability of the primer set for accurate detection of *K. pneumoniae*.

The LoD obtained with the designed primers was 1.5 ×10^4^ CFU/mL, which is consistent with other reports with LoDs of 10^4^ (Vergara et al., 2020) and 10^5^ CFU/mL (Qiu et al., 2022). However, some studies have reported LoDs near or below 10 CFU/mL (Banerjee et al., 2024; Bermúdez-Fornos et al., 2025).

Accurate diagnosis should not rely solely on rapid tests like the one proposed, but must consider the patient’s clinical context. Interpreting results alongside estimated bacterial load can help distinguish true infection from colonisation as higher concentrations are generally seen during active infections due to failed immune regulatory mechanisms (Beisswenger et al., 2009; Leshem et al., 2020; Woelfel et al., 2024).

Since molecular tests amplify faster at higher target concentrations (De Arcos-Jiménez et al., 2025), we proposed that the time to positivity in our reaction could provide an additional indicator to differentiate infection from colonisation in positive cases.

In our study, the absence of a positive signal at 45 min for *S. pneumoniae*, *S. aureus*, and at 50 min for *H. influenzae* may suggest bacterial concentrations below 10^5^ CFU/mL. This threshold is relevant for several reasons.

Various studies reported high *S. pneumoniae* loads in confirmed pneumonia cases (Dubot-Pérès et al., 2025) often exceeding 10^5^ CFU/mL in the nasopharynx (Vu et al., 2011) with 10^6^ CFU/mL suggested as a threshold to distinguish infection from colonisation (Brotons et al., 2017; Haddar et al., 2020). Similarly, *S. aureus* loads tend to be higher during active infection, with 10^5^ CFU/mL proposed as a diagnostic cut-off for methicillin-resistant *S. aureus* (MRSA) (Kwon et al., 2012). Other studies found pneumonia cases averaging 10^7^ copies/mL, while colonised patients remain below 10^5^ copies/mL, supporting its use to differentiate infection from colonisation (Huang et al., 2015).

*Haemophilus influenzae* loads above 10^4^ CFU/mL have been linked to active infection (Hare et al., 2018). Concentrations exceeding 10^6^ copies/mL are associated with confirmed childhood pneumonia (Park et al., 2017) and thresholds of >10^5^ CFU/mL may help indicate an ongoing infectious process (Kais et al., 2006).

Considering that, bacterial loads above 10^5^ CFU/mL are associated with active infections, for *S. pneumoniae* and *S. aureus*, a cut-off time of 45 min could help differentiate colonisation from true infection in our LAMP reaction. The cut-off point for *H. influenzae* should be set at 50 min, based on the association of loads above 10^4^ CFU/mL with active infections. Therefore, the time to positivity in our reaction may serve as a useful marker to distinguish between colonisation and infection. The use of bacterial suspension provides a controlled framework to assess the assay; however, validation with clinical samples remains necessary to confirm its relevance in real clinical settings.

Evaluation of the LAMP assay with clinical samples showed that the reaction can be successfully performed even with templates exhibiting variability in composition and physical characteristics. While the study primarily aimed at standardising the detection technique rather than a full clinical validation, it was important to confirm applicability to real samples, hence the limited number of specimens tested. Interestingly, in a couple of cases, LAMP detected an additional bacterium beyond that identified by culture or PCR, suggesting higher sensitivity and the ability to detect bacteria present at low load or affected by prior empirical treatment (Bouzada et al., 2025).

As previously mentioned, childhood pneumonia can be caused not only by bacteria but also by viruses, including RSV, HRV, hMPV, and influenza viruses, and others. In some cases, fungal pathogens like *P. jirovecii* may be responsible (Pneumonia in children, 2022). However, the present study focuses exclusively on bacterial detection, as these pathogens can cause more severe infections in the paediatric population (Nascimento-Carvalho et al., 2016; Nathan et al., 2020). Future adaptations and improvements of the proposed technique could include non-bacterial pathogens or even combined panels, given the potential of coinfections.

The LAMP technology described here meets most REASSURED criteria (real-time connectivity, ease of specimen collection, affordable, sensitive, specific, user-friendly, rapid and robust, equipment-free or simple, deliverable) (Land et al., 2019). However, an important limitation of this study is the need for further validation using clinical samples before confident diagnostic application.

### Conclusion

The proposed LAMP reaction shows strong potential as a simple and effective tool for detecting the main pneumonia-causing bacteria in children, with results that can be easily interpreted by the naked eye. This technique could significantly improve the diagnosis of childhood pneumonia, particularly in high-incidence settings, pending further clinical validation.

## Data Availability

The original contributions presented in the study are included in the article/Supplementary material, further inquiries can be directed to the corresponding author.
