# Supplementary material for: Development and optimization of an easy to interpret loop-mediated isothermal amplification (LAMP) assay for the identification of bacterial pathogens causing childhood pneumonia
Source: Front Microbiol. 2026 Mar 2;17:1748456. doi: 10.3389/fmicb.2026.1748456 (PMC12989572; doi:10.3389/fmicb.2026.1748456)
Supplement: Supplementary file 1 [file Data_Sheet_1.pdf]

Supplementary Material

Supplementary table 1. Compounds and concentrations used in the standardisation of the LAMP reaction

Compound	Reagent	Final Concentration
Isothermal Amplification Buffer	Tris-HCl	20 mM
	$(NH_4)2SO_4$	10 mM
	KCl	50 mM
	$MgSO_4$	2 mM
	Tween 20	0.1%
$MgSO_4$ solution 100 mM	$MgSO_4$	Variable [a]
Betaine Solution	Betaine	0.8 M
Deoxynucleotide (dNTP) Solution Mix	dATP, dTTP, dGTP, dCTP	1.4 mM each
LAMP Primers [b]	F3	0.2 µM
	B3	0.2 µM
	FIP	0.8 µM
	BIP	0.8 µM
	LB	0.4 µM
	LF	0.4 µM
Bst 2.0 Warm Start	Bst enzyme	8 U

Indicator reagent [c]	Variable	Variable
Sample [d]	Target DNA	Variable
Total reaction volume	25 µL	
Seal of mineral oil [e]	15 µL	

[a] The $MgSO_4$ concentration, varied according to the fluorescent dye tested.

[b] Primers for *S. pneumoniae* detection

[c] Different dyes were used to differentiate negative from positive LAMP reactions.

[d] Bacterial suspension of *S. pneumoniae* ATCC 49619 at 0.5 McFarland.

[e] To prevent contaminations

Supplementary table 2. Analysed calcein-Mn^{2+} and Mg^{2+} concentrations.

Test	Calcein (mM)	Mn^{2+} (mM)	Mg^{2+} (mM)	[Mn^{2+}: Mg^{2+}]
Tested calcein-Mn^{2+} combinations	0.05	1.4	8	-
	0.05	1.6	8	-
	0.05	1.8	8	-
	0.025	0.5	8	-
	0.05	0.5	8	-
	0.025	0.5	8	-
	0.025	0.6	8	-
	0.025	0.7	8	-
	0.025	0.8	8	-
	0.025	0.9	8	-

Mn^{2+}: Mg^{2+} ratios	0.025	0.5	5.5	[1:11]
	0.025	0.6	5.5	[1:9.1]
	0.025	0.7	5.5	[1:7.8]
	0.025	0.8	5.5	[1:6.8]
	0.025	0.9	5.5	[1:6.1]
	0.025	1	5.5	[1:5.5]
	0.025	1.1	5.5	[1:5]
	0.025	1.375	5.5	[1:4]

[a]Using different concentrations of *S. pneumoniae* ATCC 49619

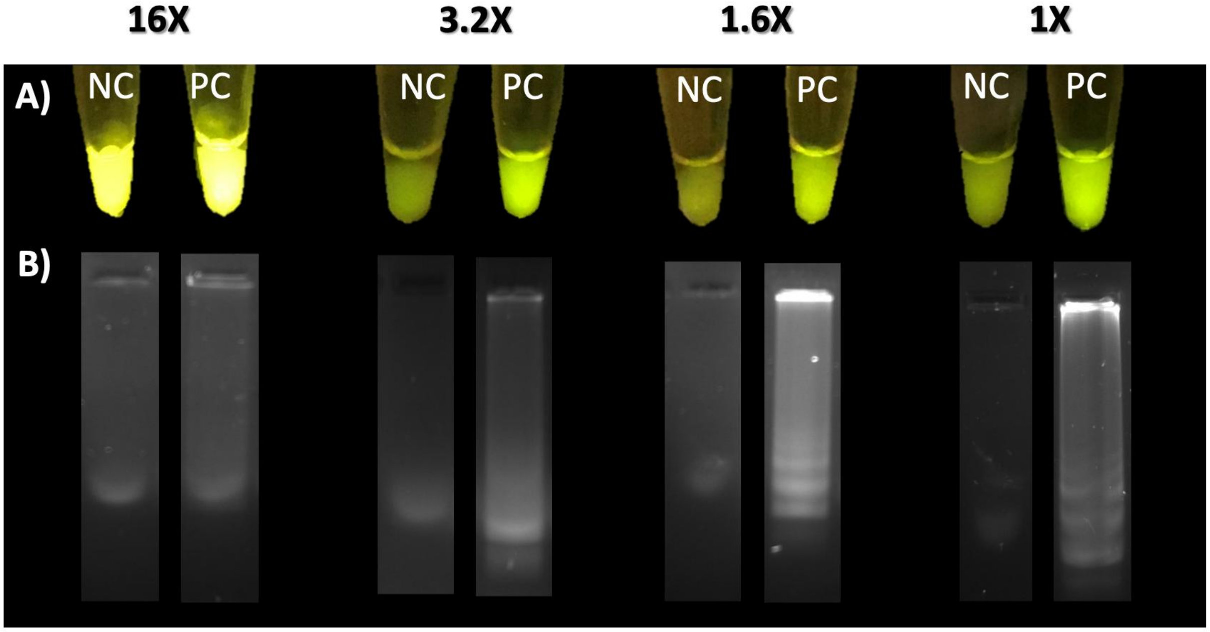

Supplementary Figure 1. Use of SYBR Safe at different concentrations as fluorescent dye in the LAMP reaction. A). Various concentrations of SYBR Safe were evaluated in the LAMP reaction. A background fluorescent signal was observed across all concentrations including in the negative

controls. Visually, at concentrations of 3.2X, 1.6X and 1X, where amplification occurred, a slight differentiation between positive and negative reactions could be detected, although the difference was not entirely clear. B). Amplification was confirmed by electrophoresis on a 2% agarose gel.

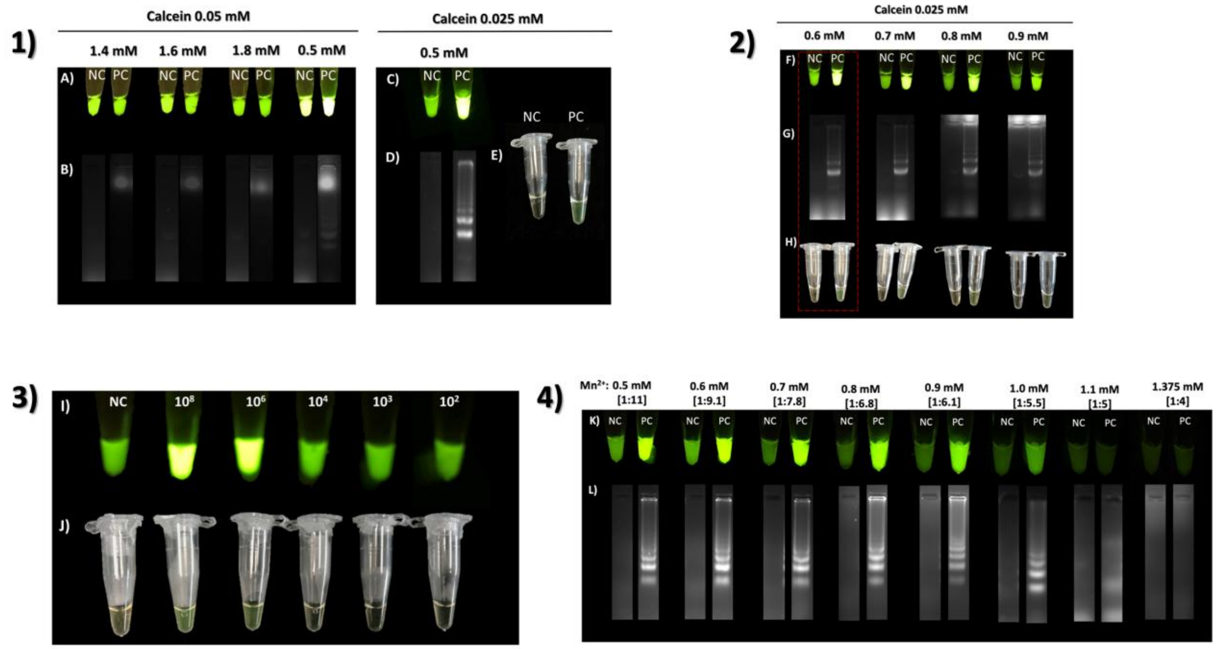

Supplementary Figure 2. Calcein combined with Mn2+ as a fluorescent dye in the LAMP reaction. 1. Different concentrations of calcein and Mn^{2+} were tested to determine the optimal conditions for distinguishing positive and negative reactions by fluorescence (A and C), confirmed by 2% agarose gel electrophoresis (B and D) and by visible color differences in the reaction tubes (E). The clearest differentiation between a positive and a negative test was obtained with calcein at 0.025 mM and Mn^{2+} at 0.5 mM, where the positive tube showed a faint greenish hue these concentrations the reaction solution is slightly green in the positive tube absent in the negative control. **2.** Fluorescence was further evaluated using 0.025 mM calcein with various Mn2+ concentrations (F), selecting 0.6 mM as optimal. Amplification was confirmed in each case by electrophoresis (G). And visible color changes matched fluorescence observations (H). **3.** Using 0.025 mM calcein and 0.06 mM of Mn^{2+} at 0.06 mM for the detection of S. pneumoniae. An increase inf fluorescence insensitivity (I) and a visible change in the color (J) of the reaction solution were only evident at higher bacterial concentrations. Only at high bacterial concentrations is an increase in the fluorescent signal and a change in the color of the reaction solution observed. **4.** Fluorescence variations were observed when different [Mg^{2+}: Mn^{2+}] ratios were tested using 0.025 mM calcein in the reaction (K). Notably, when Mn^{2+} concentration exceeded 1 mM, amplification was no longer detected, indicating an inhibitory effect (L). NC: Negative control. PC: Positive control

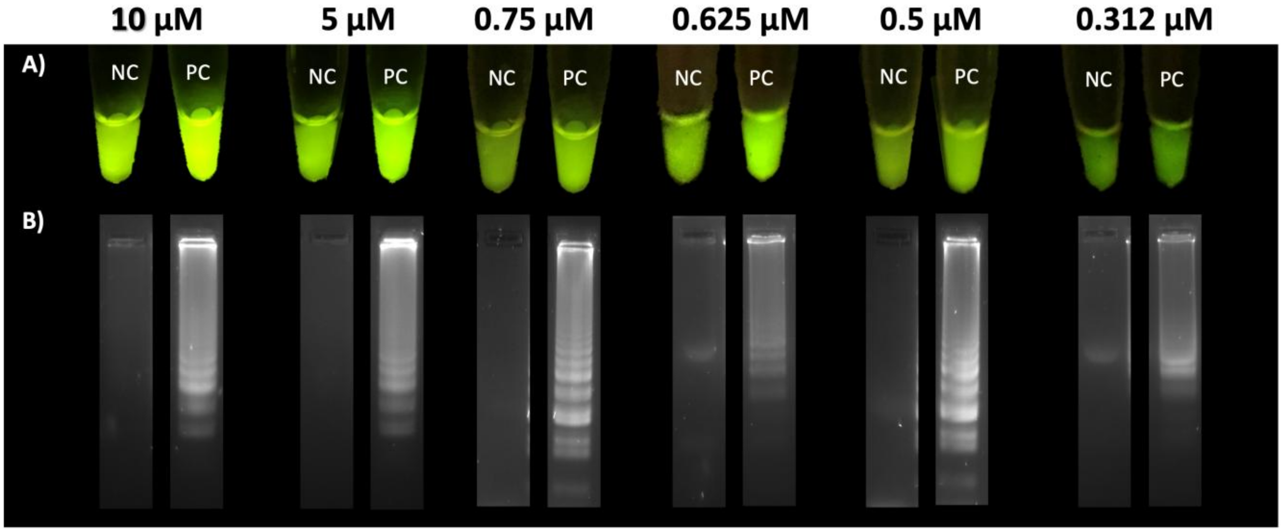

Supplementary Figure 3. Evaluation of SYTO 9 at different concentrations to differentiate between positive and negative LAMP reactions. While higher concentrations of SYTO 9 produced more intense fluorescence in both positive and negative controls, however, the distinction between them remained unclear (A). Amplification was confirmed by 2% agarose gel electrophoresis (B). NC: Negative control. PC: Positive control.

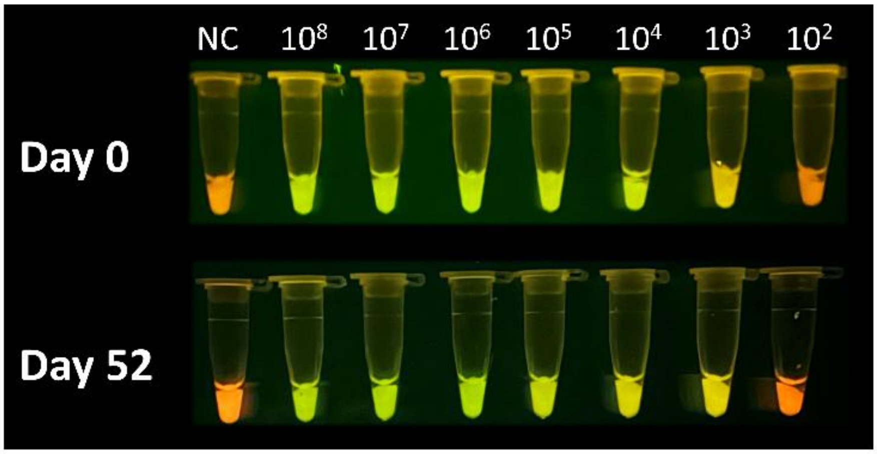

Supplementary Figure 4. Duration of the fluorescent signals emitted by HNB and SYTO 9 in the LAMP reaction. The fluorescent signals from HNB and SYTO 9 in the reaction tubes remain stable over extended periods. Over time, the green fluorescence in positive tubes gradually diminishes, while the reddish hue in negative tubes becomes more pronounced.

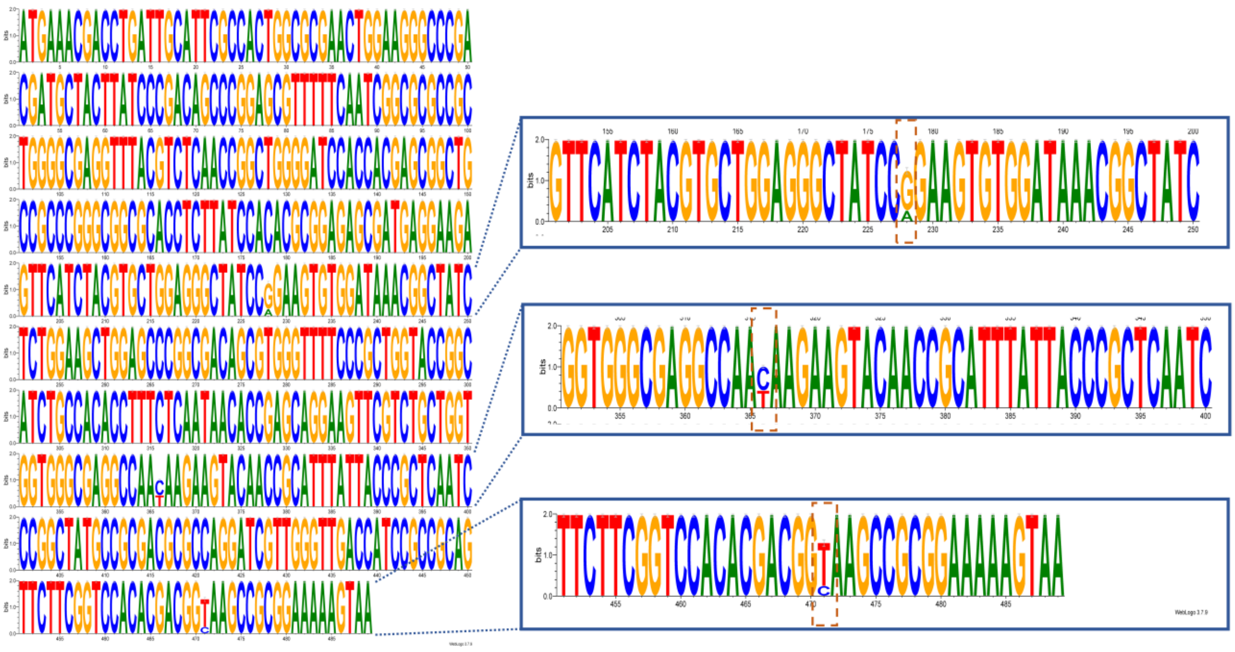

Supplementary Figure 5. Sequence conservation of the *khe* gene in *K. pneumoniae*. The target gene selected for detecting *K. pneumoniae* is a highly conserved sequence within this species. Only three base variations were identified at positions 228 (G for A), 366 (C for T) and 471 (T for C).

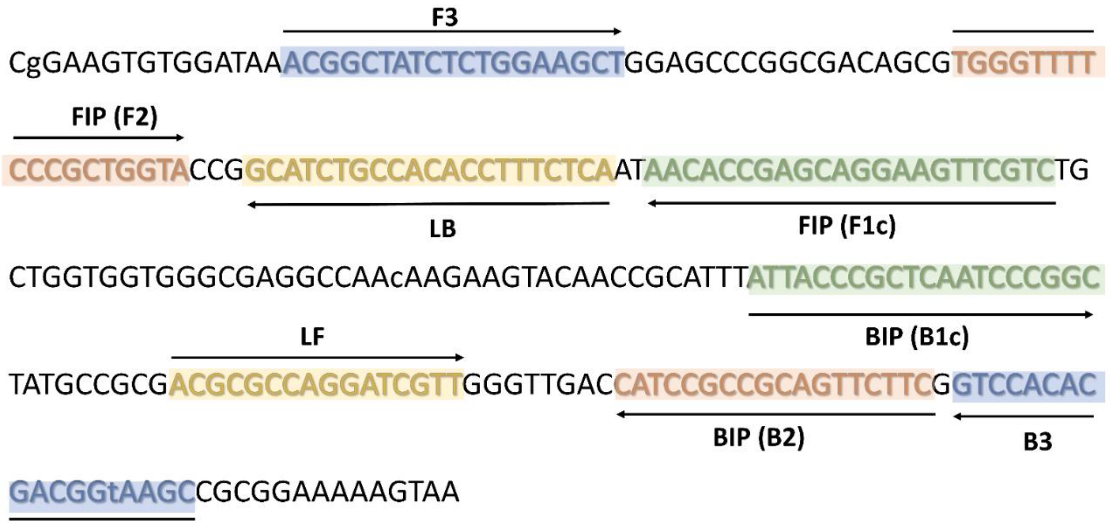

Supplementary Figure 6. Primers designed for *K. pneumoniae* detection and their target region within the *khe* gene sequence. The full consensus sequence of the *khe* gene was obtained by aligning

502 sequences. The binding sites for each primer in the designed set are indicated in the sequence with arrows showing their 5' to 3' direction.

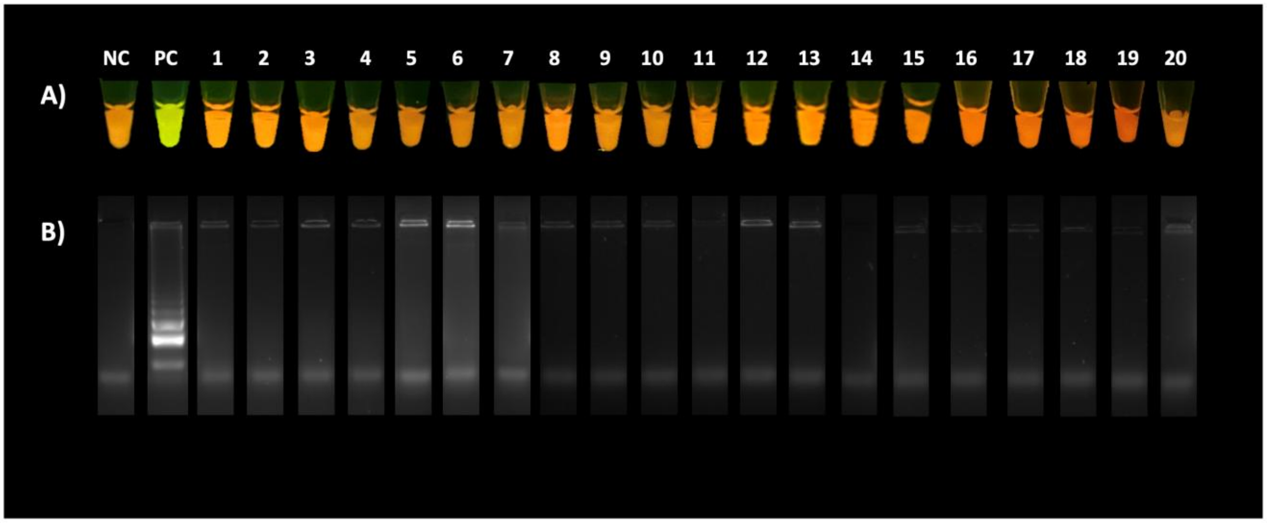

Supplementary Figure 7. Specificity of Kpne-AMT primers. The primers set was tested against different bacterial species to detect potential cross-reactivity. Visually, amplification was observed only in the positive control *(K. pneumoniae ATCC 13883)* (A). This result was confirmed by 2% agarose gel electrophoresis. NC: Negative control. PC: Positive control. 1: *S. pneumoniae* ATCC 49619. 2: *S. aureus* ATCC 25923, 3: *H. influenzae* ATCC 49766. 4: *M. pneumoniae*. 5: *S. aureus ATCC* 29213. 6: *Escherichia coli* NCTC 13846. 7: *E. coli* ATCC 25922. 8: *Acinetobacter baumannii* ATCC 17978. 9: *A. baumannii* ATCC 19606. 10: *Proteus mirabilis.* 11: *Aeromonas hydrophila.* 12: *Pseudomonas aeruginosa.* 13: *A. nosocomialis.* 14: *A. junii.* 15: *A. pittii.* 16: *Salmonella* Enterica. 17: *Serratia marcensens*, 18: *Enterobacter cloacae.* 19: *E. asburiae* and 20: *K. variicola***.**

	Sample ID	LAMP identificación	Fluorescence
			Sp Sa Hi Kp Mp IC
S. pneumoniae	4-STD	*S. pneumoniae*	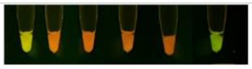
	5-STD	*S. pneumoniae/ H. influenzae*	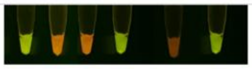
	13-STD	*S. pneumoniae/ H. influenzae*	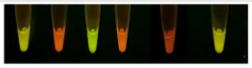
	37-STD	*S. pneumoniae*	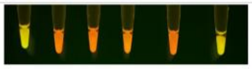
S. aureus	2-STD	*S. aureus*	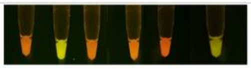
	33-STD	*S. aureus*	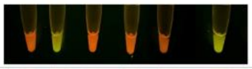
	40-STD	*S. aureus*	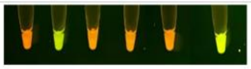
	49-STD	*S. aureus*	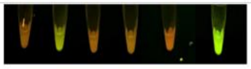
	67-STD	*S. aureus*	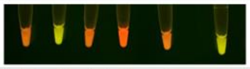
	72-STD	*S. aureus*	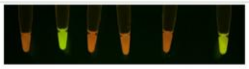
H. influenzae	13-STD	*S. pneumoniae/ H. influenzae*	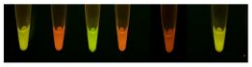
	16-STD	*S. pneumoniae/ H. influenzae*	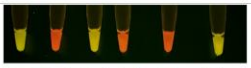
	19-STD	*H. influenzae*	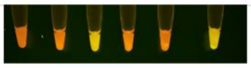
K. pneumoniae	43-STD	*K. pneumoniae*	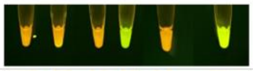
	64-STD	*K. pneumoniae*	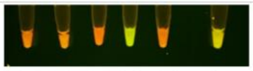
	79-STD	*K. pneumoniae*	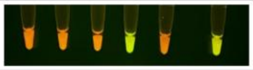
	83-STD	*K. pneumoniae*	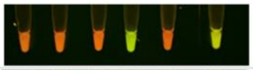
	91-STD	*K. pneumoniae*	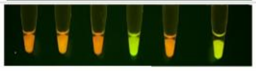
Negatives	23-STD	Negative	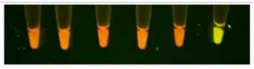
	47-STD	Negative	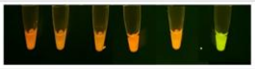
	54-STD	Negative	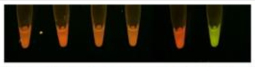
	62-STD	Negative	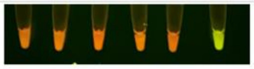
	66-STD	Negative	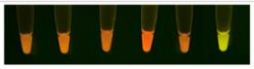

Supplementary Figure 8. Visual discrimination of positive and negative LAMP reactions using endpoint fluorescence in clinical samples. For each clinical sample analysed, photographs of the fluorescence in each tube for all panel bacteria are shown for the analysed clinical samples. Positive tubes display green-yellow fluorescence, while negative tubes showed a reddish fluorescence, demonstrating the robustness of the visual readout.

Sp: *S. pneumoniae*; **Sa**: *S. aureus*; **Hi**: *H. influenzae*; **Kp**: *K. pneumoniae*; **Mp**: *M. pneumoniae*; **IC**: Inhibition control.
